# Towards natural language question generation for the validation of ontologies and mappings

**DOI:** 10.1186/s13326-016-0089-6

**Published:** 2016-08-08

**Authors:** Asma Ben Abacha, Julio Cesar Dos Reis, Yassine Mrabet, Cédric Pruski, Marcos Da Silveira

**Affiliations:** 1 Luxembourg Institute of Science and Technology (LIST), Esch-sur-Alzette, Luxembourg; 2 Institute of Computing, University of Campinas, Campinas, Brazil

**Keywords:** Ontology validation, Mapping validation, Question generation, Knowledge management

## Abstract

**Background:**

The increasing number of open-access ontologies and their key role in several applications such as decision-support systems highlight the importance of their validation. Human expertise is crucial for the validation of ontologies from a domain point-of-view. However, the growing number of ontologies and their fast evolution over time make manual validation challenging.

**Methods:**

We propose a novel semi-automatic approach based on the generation of natural language (NL) questions to support the validation of ontologies and their evolution. The proposed approach includes the automatic generation, factorization and ordering of NL questions from medical ontologies. The final validation and correction is performed by submitting these questions to domain experts and automatically analyzing their feedback. We also propose a second approach for the validation of mappings impacted by ontology changes. The method exploits the context of the changes to propose correction alternatives presented as Multiple Choice Questions.

**Results:**

This research provides a question optimization strategy to maximize the validation of ontology entities with a reduced number of questions. We evaluate our approach for the validation of three medical ontologies. We also evaluate the feasibility and efficiency of our mappings validation approach in the context of ontology evolution. These experiments are performed with different versions of SNOMED-CT and ICD9.

**Conclusions:**

The obtained experimental results suggest the feasibility and adequacy of our approach to support the validation of interconnected and evolving ontologies. Results also suggest that taking into account RDFS and OWL entailment helps reducing the number of questions and validation time. The application of our approach to validate mapping evolution also shows the difficulty of adapting mapping evolution over time and highlights the importance of semi-automatic validation.

## Introduction

An ontology can be defined as a formal, explicit specification of a shared conceptualization [1] that can play a key role in many different applications [2]. In the medical domain, ontologies are becoming popular to represent clinical knowledge. Several ontologies became a *de facto* standard in the domain (e.g., SNOMED CT^1^, NCI^2^). As multiple ontologies can describe the same domain, semantic mappings are often defined to link ontology elements that refer to the same real-world entity but belong to different domain-related ontologies [3]. These links play a key role for systems interoperability tasks as they allow them to reconcile data annotated using different ontologies [4].

New ontologies and mappings are frequently published and updated on the Web. For instance, Bioportal^3^ is a biomedical ontology repository where more than 350 different ontologies are published and maintained. CISMeF^4^ is another example of how biomedical ontologies can be used to retrieve relevant information on the Web. The successful application of ontologies brings new challenges related to their construction, maintenance and validation. Natural Language Processing (NLP) techniques have been extensively used to retrieve and extract information from textual sources in order to automatically identify concepts, instances, and relations used in a specific domain. This led to the rapid growth of biomedical ontologies, and consequently, to an increasing need of content validation.

Erroneous facts can be included in ontologies because of several factors; including automated concept and relation extraction methods, disagreements between different human actors involved in ontology design, and ontology evolution. The ontology validation process can target different aspects depending on the requirements of validation [5]: e.g., human understanding, logical consistency, modeling issues, ontology language specification, real-world representation and semantic applications (a summary of specific techniques for ontology evaluation can be found in [6]).

While a wide range of approaches tackled logical consistency, few approaches considered the validation of the conceptualization itself from a domain point of view. In this article, we propose a semi-automatic approach for the conceptual validation of ontologies and their mappings. By conceptual validation we refer to the assessment of whether a given fact is true or false with regards to real-world knowledge: i.e., is the real-world model compliant with the formal model (ontology)? [7]. We propose a semi-automatic approach to conceptual validation based on the automatic generation of natural language questions and the processing of experts answers to these questions. We propose an optimization method to reduce the number of questions required to validate a given ontology using RDFS and OWL entailment [8].

Our second goal is to validate mapping relations between different ontologies (e.g., equivalentClass, subClassOf, equivalentProperty, subPropertyOf). We particularly focus on the context of evolving ontologies and the validation of automatically-generated mapping adaptations. We propose an adapted semi-automatic validation approach based on the automatic generation of natural language questions. When the expert invalidates a given mapping, Multiple-Choice Question (MCQ) are automatically generated to propose correction alternatives from the ontology itself [9].

Our contributions can be summarized as follows:

- We propose a semi-automatic approach to simplify the human intervention during the validation of an existing ontology. The proposed technique automatically generates well-formulated questions from the target ontology using pattern-based methods. The introduced patterns are instantiated with ontology labels to generate Boolean questions, which are submitted to domain experts. The answers returned by the experts are used to prune a subset of the remaining questions using inverse RDFS entailment. To this end, the initial question sets are ranked according to their impact on the remaining questions following RDFS entailment rules. This approach can also be applied to subsets of ontologies corresponding to their evolution (i.e., new and modified facts).
- We extend our approach to address the problem of mapping validation. We propose a novel approach to support human experts to validate recommended modifications in mappings affected by ontology evolution. We investigate techniques to generate MCQ that allow suggesting new decisions in the mapping modification process. The proposed approach analyses both the old and the updated context of concepts to propose alternative choices if the initial adapted mappings are invalidated by the experts.
- We experimentally assess our approaches conducting evaluations using real-world biomedical ontologies and mappings established between them. We measure different aspects to observe the quality and effectiveness of the questions and the defined approaches. Our results show innovative findings regarding the way that questions are generated and their relevance for the validation of ontologies and mappings.

We present and discuss research work related to question generation and the validation of ontologies and mappings in Section “Background”. In Section “Methods” we present our approaches for the validation of ontologies (Section “Ontology validation method”) and their mappings (Section “Mapping validation method”). Our experiments and results are detailed and discussed in Sections “Experimental evaluation” and “Discussion and Future work”.

## Background

In this section, we review existing techniques related to ontology evaluation (e.g., ontology verbalization) and methods related to the validation of ontologies and mappings. We also present the original aspects of our approach with regard to related works.

### Ontology evaluation criteria

Initial approaches tackling the quality of ontology contents emphasized on statistical aspects such as the number of classes, the number of properties or the number of leaf classes [10]. While these numbers reveal some information about the complexity of an ontology, they do not cover other aspects of validation as discussed in Section “Introduction”.

In their work in the PERTOMED project, Baneyx and Charlet [11] introduced several relevant criteria for the evaluation of ontology quality at various moments of its life-time (i.e., construction, evolution and maintenance) with a particular focus on the biomedical domain and its specifics. Some of the criteria deal with the structural and logical aspects of the ontology, while others tackle the conceptualization of the represented domain. Among those related to the conceptualization, they discuss the ontological commitment as essential. They advocate that when designing an ontology, a minimal number of hypotheses must be assumed to represent the domain. The authors also stress the usability of the ontology as well as its ability to fulfill the set of requirements it has been designed for.

Stvilia [12] defines a model with twelve different criteria to evaluate the quality of an ontology. He also considers statistical criteria which exploit explicitly-defined ontological elements, including: the number of classes or properties, subjective aspects like semantics and structural consistency. He also considers volatility as a new criterion to evaluate the duration for which an ontology remains valid by measuring the period of time elapsed between two successive updates. Therefore he takes into account, to a certain extent, the evolution of the considered ontology. As noted by Baneyx and Charlet [11], this work also considers the usability of an ontology by counting the number of applications that are using it.

Djedidi and Aufaure [13] proposed an approach to assess the quality of an OWL ontology at evolution time. They proposed a set of quality criteria dealing with complexity, cohesion (e.g., average number of connected components), conceptualization (e.g., average number of object properties per classes), abstraction (e.g., maximum number of classes between the root and the leaves of the ontology), completeness and comprehension (i.e., number of annotated classes or individuals). Nevertheless, the proposed approach is clearly dependent on the OWL model, since the implementation of the metrics relies on OWL primitives.

Sabou and Fernandez [6] introduced two other dimensions to consider when evaluating ontologies. The first one consists in finding relevant criteria for the selection of an existing ontology, instead of creating a new ontology from scratch. This makes sense because of the large number of available ontologies through the Web. The second criterion deals with the modularity of an ontology. They proposed to evaluate modules that require combination for a given purpose, or for a particular application, to decide the relevance of an ontology.

More recently, Rico et al. [14] presented the *OntoQualitas* framework. In this work, no new type of criterion addressing the quality of an ontology has been introduced, but the metrics to calculate them have been improved and refined. The authors also provided a concrete case study to assess the framework.

### Question generation and verbalization of ontology content

Several efforts have addressed the automatic generation of NL questions^5^. Most of them focused on the generation of questions from text (text-to-question task) [15]. The question generation process can rely on manual patterns [16] and/or on statistical techniques [17].

*AUTOQUEST* [15] stands for one of the first question generation systems proposed to assess text understanding. More recently, Heilman [17] proposed to generate WH questions from text, relying on hand-crafted transformation patterns and a statistical ranking model. For a related e-learning task, Liu et al. [16] proposed *G-Asks*, an automatic question generation system. To support learning through writing, *G-Asks* generates specific questions using manual patterns that are associated with different question types. Mitkov and Ha [18] tackled the generation of multi-choice questions from instructional documents. The main proposed process employs NLP techniques for domain term extraction and shallow parsing. Their work also defined hand-crafted transformational rules to generate the questions from declarative sentences with minimal modification to the original words.

The current volume of dense ontologies requires novel techniques to guarantee the semantic precision of the contents. Papasalouros et al. [19] suggested an automatic approach to generate MCQs from ontologies that remains independent from linguistic resources and domain-specific constraints. They proposed ontology-level strategies according to the basic RDFS types and primitives (e.g., classes, properties and *subClassOf* axioms). The approach defined these strategies to produce distractors in the scope of computer-assisted assessment. However, they do not explore advanced NL generation methods. For example, the same stem, “*Choose the correct sentence*" is used in all questions. Similarly, Cubric and Tosic [20] examined the same type of ontology-related strategy for e-assessment, by defining a generic questions ontology and linking it to domain ontologies.

The *MoKI* systems designed by Pammer [21] is, to the best of our knowledge, the only work that addresses the problem of ontology validation by means of question-answering techniques. The proposal is to generate questions from the content of the ontology and submit them to domain experts in order to get their feedback, leading to the validation or modification of the underlying ontology. However, the question generation process does not integrate the fact that domain experts are rarely familiar with formal ICT languages. The questions generated by *MoKI* look very similar to description logic formulas hardly understandable by experts. Hence, the outcome of the proposed system still require a substantial intervention of ICT experts to support domain experts through the validation process.

In another application, Teitsma et al. [22] presented an ontology-based generation approach for situation determination. A traffic accident ontology and two databases on accidents were used to: (i) generate questions from infons (sets of field values describing the situation; such as weather or injuries) and (ii) get validation answers from human observers who watched video scenes describing the target situations. They used rule-based logical optimization techniques (e.g., if the observers validated ’rain’ for weather the system does not ask if the road is ’wet’). However, this work does not provide details on the NL level of the generation process, which seems adhoc with respect to the concepts of the selected domain ontologies.

### Ontology validation

As discussed above, a significant amount of work tackled the study and proposition of a set of metrics dealing with the quality of ontologies. These metrics are based on measurable properties such as the number of classes, individuals and properties. While it can be argued that such metrics can give a relevant evaluation of the quality of an ontology, they cannot be used to validate the ontology content, i.e., an ontology with a “good” quality can contain more erroneous facts that an ontology with an estimated “lower” quality.

Gangemi et al. introduced a model for evaluating and validating ontologies [23]. Based on a meta-ontology called *O*^2^ and semiotics, the authors propose to evaluate ontologies by considering structural, functional and usability-profiling measures. The validation is then complemented with an ontology called *oQual* aiming at providing necessary criteria to select an ontology to answer a particular need.

Köhler et al. provided a rule-based methodology (*i.e.*, a set of conventions) to establish well-defined labels for *Gene Ontology (GO)* concepts [24]. The rules aim at avoiding circular definitions (*i.e.,* term of the label that are also in the definition of the considered concept) and obscure language (*i.e.,* labels of concepts must be understood by non expert persons). Although interesting, this work can hardly be applied to other ontologies because GO labels are very domain specific. They are not only built on linguistic aspects, but they use a lot of alphanumeric symbols to denote gene and proteins.

A similar argument is used by Verspoor et al. [25]. The authors proposed a methodology to classify medical terms that denote the same meaning but use different linguistic conventions in order to standardize them. Such kind of approaches addresses an important aspect which is the choice of the terminology to describe ontological elements. However, elements that are implicitly defined (e.g., concepts that are logically defined by inference) are not standardized.

Dimitrova et al. designed the *ROO* tool for supporting domain experts designing OWL ontologies [26]. This provides a controlled language interface and offers systematic guidance throughout the whole ontology construction process with an aim of optimizing the quality of the resulting ontology. However, nowadays ontologies are rather built automatically from the textual content of relevant documents or data [27, 28], or rather slightly modified by virtue of knowledge evolution. Accordingly domain experts are mostly involved in the validation phase and less and less from the beginning of the ontology life cycle.

In their work [29], vor der Bruck and Stenzhorn described a method to validate ontologies using an automatic theorem prover and *MultiNet* axioms. To this end, the authors focused on the logical structure and ignored the conceptualization part. Therefore, their method requires formal ontologies expressed in logic-based languages, which is not always the case in the biomedical domain. The system implementing the proposed algorithm, is accompanied with a user friendly software interface to speed up the fixing of the detected erroneous axioms facilitating users intervention.

More recently, Poveda-villalón et al. [5] proposed the *OOPS!* system. This consists of detecting pre-defined anomalies or bad practices in ontologies to enhance their quality. However, the real-world representation dimension is neglected in this approach, which refers to how accurately the ontology represents the domain intended for modelling. This is left to the discretion of domain experts.

Other families of approaches have been proposed addressing the validation of the domain-conceptualization side. Some of them emphasize on user interface development to better present large amount of (structured) data without overwhelming users [30] to support the validation effort. Other research work promotes the use of NLP techniques to better involve domain experts in the ontology validation process [21].

### Mapping validation

Similar to ontologies, previous studies have revealed the real difficulties and importance of validating mappings and involving human experts in the process [31]. Mapping revision refers to a method aiming to identify and repair invalid mappings that can be explored for mapping validation. Existing techniques may detect the invalid mappings at ontology evolution time. Meilicke et al. [32] proposed an automatic mapping debugging between expressive ontologies eliminating inconsistencies, caused by erroneous mappings, through logical diagnostic reasoning. Mapping revision still demands logically expressive ontologies. This motivates further research on alternative methods to validate the adaptation of mappings. Similarly, Serpeloni et al. [33] proposed a semi-automatic process to validate mappings through graph algorithms that select instances for verification.

In order to take human aspects into account, recent studies have examined the interactive aspect to validate mappings. Some approaches tackled the design of interactive tools to support the ontology mapping process with relevant visualizations [34]. Other studies proposed ontology alignment and validation based on users in community [35] and crowdsourcing [36]. However, these users are not necessarily experts in the (sub-)domain represented by the ontology.

### Positioning

Although several studies addressed recently the tasks of ontology validation, mapping validation and question generation, little attention has been given to the problem of validating ontology contents (including semantic alignments) from the perspective of domain conceptualization. In this context, our first contribution is a semi-automatic approach to reduce and simplify the human interventions required to validate ontology contents and mappings from a domain point of view. Our approach is based on the generation of boolean questions from the ontology. Expert answers to these questions are then processed automatically to validate and correct the ontology. We also use the expert feedback incrementally to prune a subset of the remaining questions using inverse RDFS entailment.

On the other hand, to the best of our knowledge, there are no studies that tackle particularly the possibility of validating ontology mappings via automatic question generation. We propose a mapping validation approach that tackles the special characteristics of modifications in mappings over time, taking the involvement of users into account. More precisely, our second contribution aims to support human experts during the mapping validation process.

## Methods

We briefly present the preliminary definitions needed to describe our approach. We first introduce the formal notions of *ontology* and *mapping* before defining the problem of conceptual validation and mapping validation (Section “Definitions and problem statement”). In the second part of this section we present our optimisation approach for the generation of boolean questions to validate ontologies (Section “Ontology validation method”) and our method to search for correction alternatives for invalid mappings (Section “Mapping validation method”).

### Definitions and problem statement

An ***ontologyO*** =(*Concepts, Relationships, Attributes*) consists of a set of *Concepts* interrelated by directed *Relationships*. We define a set of concepts of an ontology *O*_*x*_ at time *t* as $Concepts({O^{t}_{x}})=\{{C^{t}_{1}}, {C^{t}_{2}},..., {C^{t}_{n}}\}$. Each concept *C*∈*Concepts* has a unique identifier and is associated with a set of attributes *Attributes*(*C*)={*a*_1_,*a*_2_,...,*a*_*p*_} (e.g., label, definition, synonym, *etc*.). A relationship *r* ∈*Relationships* interconnects two concepts and has a specific type, e.g., ’is_a’ or ’part_of’.

The ***context of a concept*** (CT(*C*_*i*_)) in the ontology stands for the union of the sets of *super concepts* (*sup*(*C*_*i*_)), *sub concepts* (*sub*(*C*_*i*_)) and sibling concepts of *C*_*i*_ (*sib*(*C*_*i*_)), as following:

1 $$  CT(C_{i}) = sup(C_{i}) \cup sub(C_{i}) \cup sib(C_{i})  $$

where

$$ \begin{aligned} &{}sup(C_{i}) = \{C_{k}|C_{k} \in Concepts(O), C_{i} \sqsubset C_{k} \wedge C_{i} \neq C_{k} \}\\ &{}sub(C_{i}) = \{C_{k}|C_{k} \in Concepts(O), C_{k} \sqsubset C_{i}\wedge C_{i} \neq C_{k} \}\\ &{}sib(C_{i}) = \!\{C_{k}|C_{k} \!\in \!Concepts(O),\! \exists a,b s.t. C_{a} \!\in \!sup(C_{k}) and C_{a} \!\in\! sup(C_{i})\} \\ \end{aligned}  $$

where $C_{i} \sqsubset C_{k}$ means that “ *C*_*k*_ subsumes *C*_*i*_”.

An ontology mapping $M_{O_{A},O_{B}}^{t}$, established at time *t*, interlinks a set of given concepts *C*_*a*_ and *C*_*b*_ from two different ontologies *O*_*A*_/ *O*_*B*_ by so-called correspondences: $M_{O_{A},O_{B}}^{t} \!\!\!\,=\, \{(\!{C_{a}^{t}}, {C_{b}^{t}}, \mathit {semType}_{ab}^{t}, conf^{t}, status^{t}) | C_{a} \in \mathit {Concepts} (O_{A}), C_{b} \in \mathit {Concepts}(O_{B}),\mathit {confidence}\in [0,1], \mathit {semanticType} \in \{\equiv, \leq, \geq,\approx \}, \mathit {status} \!\in \! \{"\mathit {valid}", \!"\mathit {invalid}"\!,\! "\mathit {inactive}"\!, \!"\mathit {handled}", \!"to\,-\, \mathit {verify}"\}\}$

A ***correspondence***$\mathit {cor}_{C_{A},C_{B}}=(C_{A},C_{B},\mathit {confidence}, \mathit {semanticType})$ links two concepts *C*_*A*_∈*Concepts*(*O*_*A*_) and *C*_*B*_∈*Concepts*(*O*_*B*_). The *confidence* value represents the semantic similarity between *C*_*A*_ and *C*_*B*_ (indicating the confidence of their relation [3]). The higher the value, the more related are both concepts. The *semanticType* in $cor_{C_{A}, C_{B}}$ refers to the semantic relation connecting *C*_*A*_ and *C*_*B*_. We consider the following types of semantic relations: *unmappable* [ ⊥], *equivalent* [ ≡], *narrow-to-broad* [ ≤], *broad-to-narrow* [ ≥] and *overlapped* [ ≈].

The ***conceptual validation problem*** consists of defining a method to get feedback from domain experts on the correctness of ontology facts (or mappings) and interpret their answers to modify the ontology/mapping accordingly. We propose to examine question generation techniques to cope with the conceptual validation problem. Two issues have to be investigated in particular:

- How to formulate Natural Language (NL) questions that would lead to expert answers that are both relevant and computer-interpretable? (clarity problem)
- How to avoid overwhelming human experts with unnecessary questions? (optimisation problem)

We define the mapping validation problem as follows: starting from an ontology *O*_*A*_ at time *t*, noted ${O^{t}_{A}}$, and another different ontology *O*_*B*_ at time *t*, noted ${O^{t}_{B}}$, a set of correspondences exist between them $M_{{O^{t}_{A}}, {O^{t}_{B}}}$. In particular, the investigated problem consists in validating the correspondences if the ontology ${O^{t}_{A}}$ evolves to a new version $O^{t'}_{A}$ at time *t*^′^. We divide the problem by considering the evolution of only one ontology at a time (i.e., the target ontology remains unchanged).

In the following, we define our methods for ontology validation (Section “Ontology validation method”) and mapping validation (Section “Mapping validation method”).

### Ontology validation method

#### Approach overview

**Fig. 1:**
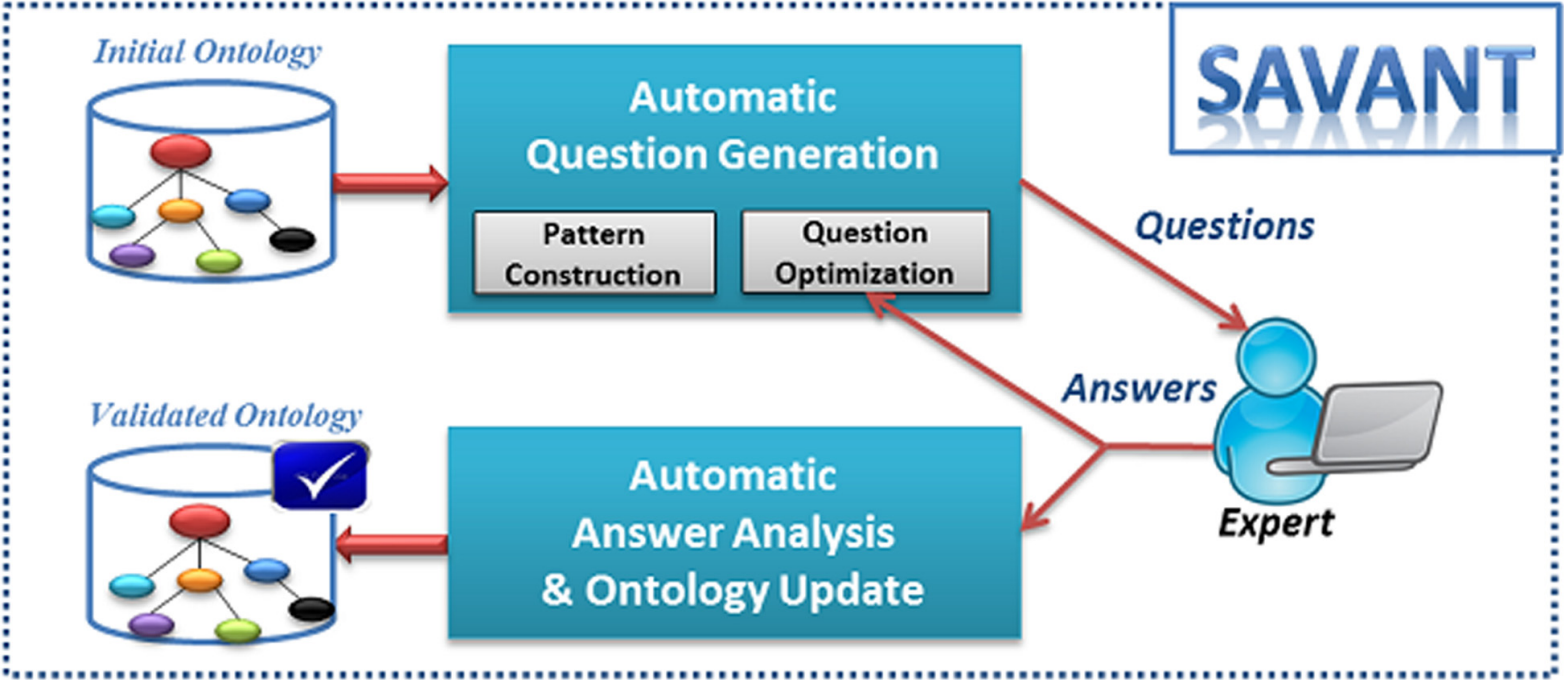
Proposed approach to ontology validation based on the automatic generation of boolean questions

We propose a semi-automatic approach based on question generation to validate ontologies. Figure 1 describes our ontology validation system, called SAVANT. The first step consists of automatically generating a list of boolean questions from the ontology under validation.

These questions are submitted to domain experts who provide an agreement decision (Yes/No) and a textual feedback. The next step consists on interpreting expert’ feedback to validate or modify the ontology. The novelty of our approach relies on the fact that manual interventions are performed only by Health Professionals (HPs), who will lead the ontology validation process. ICT experts are required only when the error cannot be solved automatically. This increases the quality of exchanges between actors and reduce errors and time consumption.

We explore the proposed approach to (i) validate ontologies constructed automatically from medical texts (e.g., clinical guidelines) and also (ii) to re-validate ontologies (constructed manually or automatically), since medical knowledge evolves quickly over time.

We focus on validating the following types of ontology statements:

- A rdfs:subClassOf B (class A is a subclass of B)
- P rdfs:subPropertyOf Q (property P is a sub-property of Q)
- P rdfs:domain D (D is the domain class for property P)
- P rdfs:range R (R is the range class for property P)
- I rdf:type A (I is an individual of class A)
- I P J (the property P links the individuals I and J)

The proposed approach uses manually constructed patterns for each kind of ontology element as described in the following section.

#### Pattern-based method for boolean question generation

We start from the hypothesis that all the elements of a medical ontology must be validated. This involves validating concepts (e.g., Substance), relations between concepts (e.g., administrated for), concept instances (e.g., activated charcoal is an instance of Manufactured Material), relations between concept instances (e.g., chest X-ray can be ordered for Chronic cough) or between concept instances and literals (e.g., “give oral activated charcoal 50 g” indicates the dose of the substance to be administrated “50 g”). These ontology elements provide the main keywords of the question patterns through the labels of concepts, relations and instances.

We constructed manually question patterns associated to each type of ontological element (5 different elements in our preliminary experimentations). A question pattern consists of a regular textual expression with the appropriate “gaps” [37]. For instance, the pattern “Is DOSE of DRUG well suited for PATIENTS having DISEASE?” is a textual pattern with 4 gaps: DOSE, DRUG, PATIENTS and DISEASE. This question pattern aims to validate a drug dose administrated to a patient having a particular disease. The singular or plural form of the verb in the expression is determined using the Stanford parser^6^. Singular is used by default if the detection is not possible, a frequent case that occurs because of the heterogeneity of ontology labels.

Table 1 presents examples of boolean-question patterns.

**Table 1:** Examples of boolean-questions patterns used for ontology validation

Question pattern	Example of instance
Does a(n) CLASS have a PROPERTY	Does an effect have a measurement method?
	Does a treatment have an administration scheme?
Is CLASS a type of CLASS?	Is statistical evidence a type of evidence?
Is SUB-PROP of a CLASS a PROP of the same CLASS?	Is primary treatment of a disease a treatment of the same disease?

#### Question optimization strategy

At this level, our main objective is to investigate a technique to build relevant questions from formalized knowledge in order to validate the maximum number of assertions with the minimum number of questions.

We propose an optimization strategy relying on the RDFS logical rules to rank the questions according to the elements that imply the more changes in the ontology.

For instance, if we have the following data:

- hasSuitedAntiobioticsType *rdf:subPropertyOf* hasTreatment
- Antibiotics *rdfs:subClassOf* Treatment
- hasSuitedAntiobioticsType *rdfs:range* Antibiotics

and the expert invalidates “Antibiotics *rdfs:subClassOf* Treatment", than the property *hasSuitedAntiobioticsType* cannot be declared as a sub-property of *hasTreatment* because the *hasSuitedAntibioticType* relation has not a common range with the property *hasTreatment*, which leads to a formal error regarding the RDFS entailment rules.

We consider all RDFS entailment rules^7^. Table 2 presents some inversed forms of these rules to show the impact of invalidating each one of the target ontology statements.

**Table 2:** Examples of ontology update rules with respect to invalidated elements used for ontology validation

NOT A rdfs:subClassOf B	⇒	NOT A rdfs:subClassOf C s.t. C rdfs:subClassOf B
NOT P rdfs:domain A	⇒	NOT P rdf:subPropertyOf Q s.t. Q rdfs:domain A
NOT I rdf:type A	⇒	NOT <I, P, J > s.t. P rdfs:domain A
		NOT <J, P, I > s.t. P rdfs:range A

This technique enables ranking questions in a manner that allows to delete some of the remaining questions if one of the RDFS entailment rules apply. This leads to the following validation order:

1. A rdfs:subClassOf B
2. P rdfs:domain D and P rdfs:range R
3. P rdfs:subPropertyOf Q
4. I rdf:type A
5. I P J

#### Answer analysis and ontology update

The second step of our approach refers to the exploitation of expert’ feedback to validate or modify the target ontology. The ontology under validation might contain concepts, individuals and relations defined between concepts or individuals.

Feedback consists of two main parts: (i) an assertion on the correctness of the target knowledge and (ii) a free textual explanation if provided^8^. In the scope of this article, we take into account ontologies that are formally-valid (with no inconsistencies) and emphasize the validation of domain conceptualization.

In this context, “Yes” answers have no impact on the ontology. The ontology is modified on the “No” answers provided by the domain experts. Invalidating an ontology element implies different impacts according to the element type.

We use the same RDFS entailment rules to update the ontology. The ontology item invalidated by the expert and the inferred invalidations are deleted from the ontology, as well as the questions associated to them.

### Mapping validation method

#### Approach overview

**Fig. 2:**
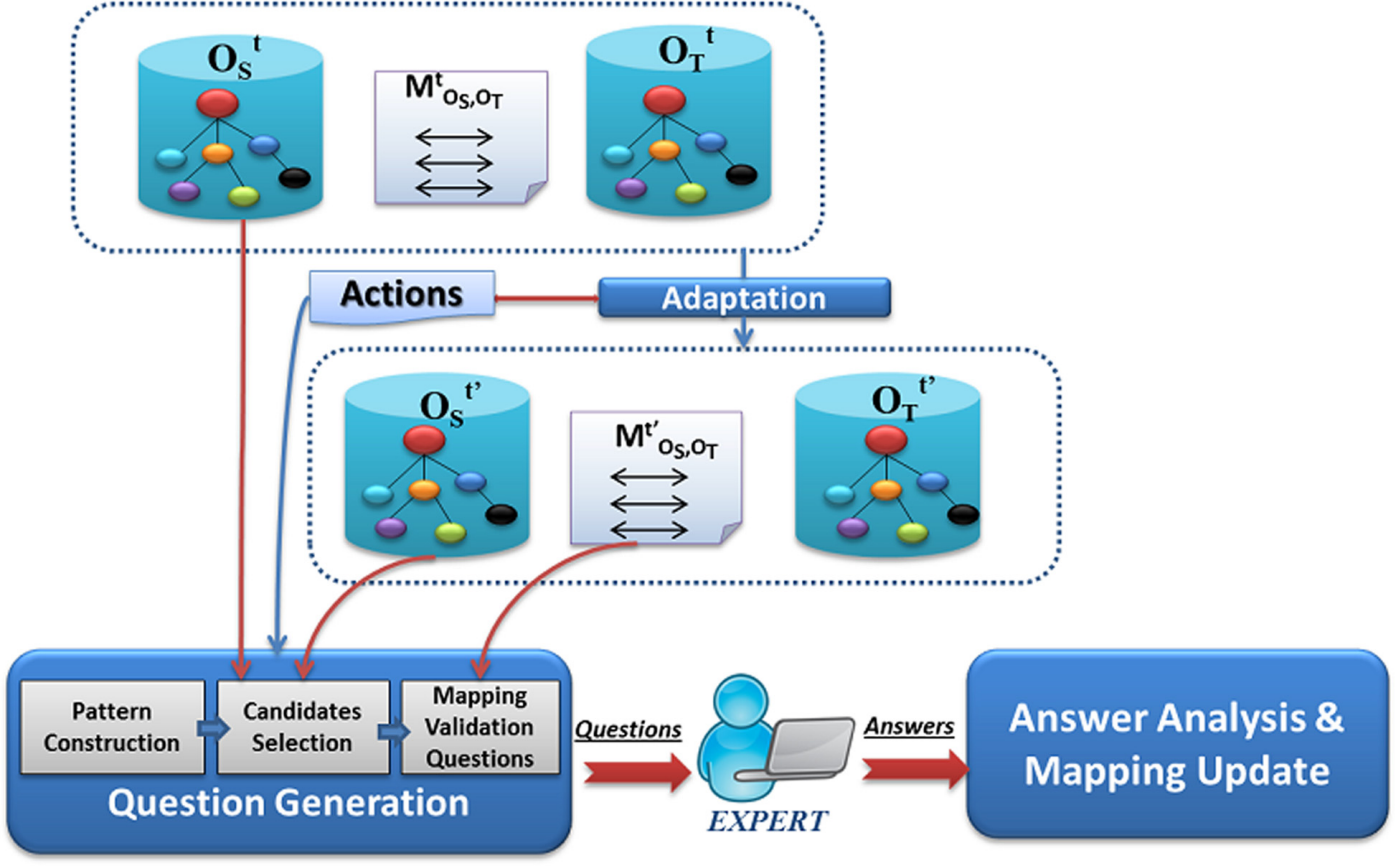
Proposed approach to validate mapping adaptation based on the automatic generation of boolean questions for new mappings and multiple choice questions for invalid mappings

**Fig. 3:**
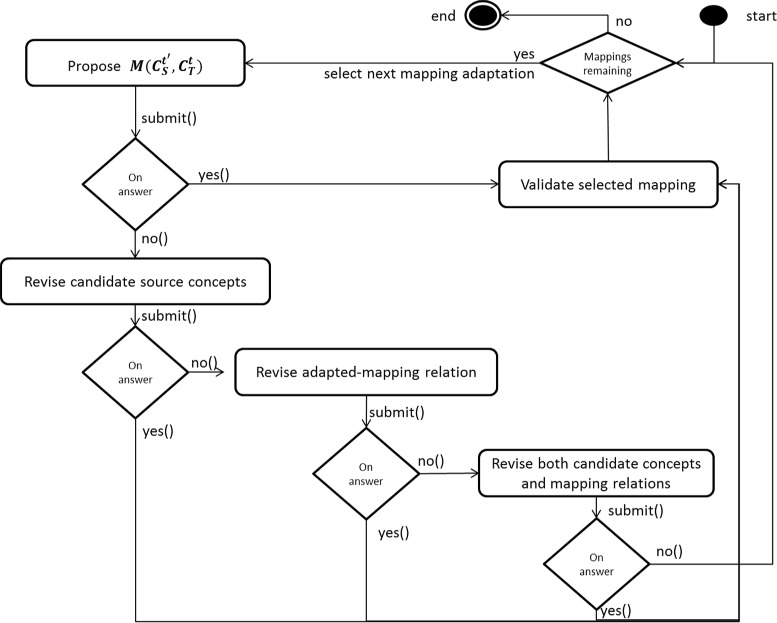
The question generation process as a state-transition diagram

We consider as input a set of adapted correspondences $M_{{O^{t'}_{A}},{O^{t}_{B}}}$. Adaptation here refers to the automatic mapping adaptation that occurs after the evolution of the source ontology ${O^{t}_{A}}$. We consider that the old mappings are correct and we want to validate only the new ones. Similarly to the ontology validation method, our approach to validate mappings relies on the generation of NL questions from the new mappings. Figure 2 describes our proposed approach for mapping validation. Figure 3 presents our method for question generation through a state transition diagram.

**Fig. 4:**
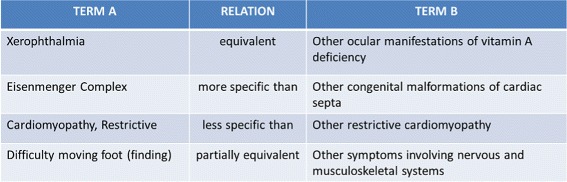
Examples of ambiguous correspondences between SNOMED-CT and ICD9CM

Table 3 presents examples of correspondences between SNOMED-CT and ICD9. Figure 4 shows examples of more or less ambiguous correspondences retrieved between the biomedical ontologies SNOMED-CT and ICD9. This selection provides concrete examples of the issues related to the heterogeneity and broadness of some concept definitions. Dealing with this problem requires to define flexible *answer types* to ensure a relevant interaction with the human validators.

**Table 3:** Examples of correspondences between SNOMED-CT and ICD9CM

Concept source label	*semanticType*	Concept target label
Intestinal diseases	equivalent to [ ≡]	Vascular disorders of intestine
Nail-Patella syndrome	more specific than [ ≤]	Congenital malformation syndromes
		predominantly involving limbs
Respiratory tract infections	less specific than [ ≥]	Acute upper respiratory infection, unspecified
Abnormality of gastric inhibitory		
peptide secretion (disorder)	partially correspond to [ ≈]	Bladder

#### STEP 1: Boolean questions

In the first step, our method translates the proposed adapted correspondences into a NL question using textual patterns associated to each relation type. Let *X* be the source concept label and *Y* be the target concept label, the main patterns are as follows:

1. (Is|Are) *X* <equivalent to >*Y*?
2. (Is|Are) *X* <more specific meaning than >*Y*?
3. (Is|Are) *X* <less specific than >*Y*?
4. Do(es) *X* <partially correspond to >*Y*?
5. *X* <cannot be matched with >*Y*?

These patterns are instantiated with the involved concepts of a correspondence. We present three instantiation examples from our dataset in the following:

1. *Are intestinal diseases equivalent to vascular disorder of intestines?*
2. *Does the Trousseau sign partially correspond to ill-defined and unknown causes of morbidity and mortality?*
3. *Is the Eisenmenger Complex more specific than other congenital malformations of cardiac septa?*

#### STEP 2: multiple choice questions

In the second and main step, negative answers trigger multiple choice questions (MCQs) that are submitted to the expert in order to detect alternative correct mappings between $C^{t'}_{a}$ (the source concept) and the concept $C^{t'}_{b}$ in target ontology $O^{t'}_{B}$. MCQ consists of (i) a problem known as the *stem* and (ii) a list of suggested *alternatives*. In our approach, we have three categories of stems/questions (*cf.* Fig. 3):

1. **Revision of***C*_*a*_. This suggests revising the source concept by candidate proposals from the new source ontology $O^{t'}_{A}$. This category preserves the semantic mapping-relation between the source concept $C^{t'}_{a}$ and the target concept $C^{t'}_{b}$, and proposes candidate concepts from $O^{t'}_{A}$ that are semantically close to the initial source concept ${C^{t}_{a}}$. In this MCQ category, we propose stems of the form: *“What concept <semanticType ><target concept >?”* corresponds to the revision of the candidate source concept, where <*semanticType*> refers to the type of mapping relation $semanticType_{ab}^{t'}$ and <target concept > consists of the label of the target concept ${C^{t}_{b}}$. For instance, an instantiation of this stem pattern is: *What concept is more specific than other restrictive cardiomyopathy?* The alternatives for this stem stand for the *top n* most semantically-close concepts to the initial source concept (e.g., *Cardiomyopath, Restrictive*). Section “Selection of alternative concepts and mapping relations” presents the selection of alternative candidate concepts.
2. **Revision of mapping relation** (*MR*). This category of questions proposes revising the type of mapping relation. More precisely, in case of a negative answer in the previous MCQ option, our method preserves the initial concept candidate $C^{t'}_{a}$ and modifies the *semanticType* of the adapted correspondence, selecting another alternative mapping relation (*cf.* Section “Selection of alternative concepts and mapping relations”). We propose stems of the form *“Choose the correct mapping relation alternative”*. The proposed alternatives are declarative sentences derived from the question patterns.
3. **Revision of both**. In case of a negative answer in the previous option, our method revises both candidate proposals and semantic relation types, aggregating both option 1 and 2 in a single multiple choice question. We formulate stems of the form *“Choose the correct source concept and relation type”* corresponding to the revision of both the candidate source concept and *semanticType* of mapping. We present alternatives for the question generation in 3 columns format, where the first column consists of the list of selected source concept alternatives, the second column presents the list of new suggested types of semantic relations and the third column contains the target concept.

We present two instantiation examples from our dataset in the following:

1. *IsOther spontaneous pneumothoraxmore specific than closed pneumothorax?*
  *Alternative source concepts:*
  *Iatrogenic pneumothorax*
  *Secondary spontaneous pneumothorax*
2. *IsGastroparesismore specific than Diabetic Gastroparesis associated 2 diabetes mellitus?*
  *Alternative source concepts:*
  *Acute dilatation of stomach*
  *Dyspepsia and other specified disorders of function of stomach*

#### Selection of alternative concepts and mapping relations

The defined approach based on MCQ (*cf.* Section “STEP 2: multiple choice questions”) requires selecting candidate concepts and different *semanticType* relations as suggested alternatives in question generation to support mapping validation over time. For this purpose, we propose an algorithm to select similar concepts to the original source concept.

#### Selection of alternative concepts

In the scope of source concept revision, we generate alternatives in MCQs by using candidate concepts from the context of the initial source concept in the ontology. We aim at combining the answers from these questions to propose re-adapting correspondences if necessary. For example, if a given correspondence between source concept ${C_{a}^{t}}$ and target concept ${C_{b}^{t}}$ is adapted, such that a concept $C^{t'}_{k} \in Concepts(O^{t'}_{A})$ replaces the original concept ${C_{a}^{t}}$, this generates an adapted correspondence at time *t*^′^ between $\phantom {\dot {i}\!}C^{t'}_{k}$ and $C_{b}^{j'} \in Concepts(O^{t'}_{B})$.

Therefore, we retrieve from the ontological context *CT* (*cf.* Eq. 1) a set of other concepts which differs from $C^{t'}_{k}$, $Candidates = \{(C^{t'}_{a_{i}}, sim_{i}) i \in \,[1..n]\}$, where $C^{t'}_{i} \in CT(C^{t'}_{a})$.

Algorithm 1 presents the designed procedure to retrieve the candidate concepts from the context, given a source concept ${C^{t}_{a}}$ of a mapping. The algorithm sorts the best *top n* candidate concepts from $CT(C^{t'}_{a})$ using a similarity measure. We use the the *bigram* similarity measure following the observations of [38] on its suitability for ontology matching tasks. For two given labels, Bigram similarity is computed as the euclidean distance, using all possible bigrams from both labels as dimensions. In our approach, we compute the similarity between pairs of comparable attributes that are selected beforehand as a parameter (e.g., the name and synonym attributes). We denote the similarity function as $simAtt({a^{t}_{i}}.value, a^{t'}_{j}.value)$ between two attribute values ${a^{t}_{i}}.value$ and $a^{t'}_{j}.value$.



Given all attributes of the original source concept, the algorithm retrieves all concepts in context *CT* at time *t*^′^. For all retrieved concepts different from the concept $C^{t'}_{k}$, to which the adapted mapping is associated, the algorithm selects their attributes and calculates the similarity between the attribute values (between attributes of the source concept and attributes of concepts in *CT*). For each candidate concept, the algorithm keeps the maximal similarity value calculated among the attributes. Finally, the algorithm sorts the top *n* retrieved candidate concepts according to the calculated similarity. We use these candidates as alternative answers in our MCQ approach, so they play a central role for the automatic generation of the questions.

#### Selection of alternative mapping relations

Revising the semantic relation *semanticType* in our question generation method demands retrieving alternatives for the second category of proposed MCQ (*cf.* Section “STEP 2: multiple choice questions”). To this end, we recover a set of semantic relations *Rel*_*alternatives*_={(*semanticType*_*i*_)*i*∈ [1..*n*]} where *semanticType*_*i*_∈{≡,≤,≥,≈} such that $semanticType_{i} \neq semanticType_{ab}^{t'}$. We use the *Rel*_*alternatives*_ to formulate the question in the revision of *MR* category. The alternative relations are proposed from the most precise one to the more general one (i.e., ≡, ≤, ≥, then ≈).

## Experimental evaluation

We selected a set of ontologies and mappings and designed a series of experiments to evaluate the proposed methods. In this article, we considered medical ontologies and mappings in English language, but our approaches can be applied to other languages as well. We present the obtained results in Sections “Experiments on ontology validation” and “Experiments on mapping validation” and discuss our findings in Section“Discussion and Future work”.

### Experiments on ontology validation

#### Materials

We tested our ontology validation approach on three different medical ontologies that cover different aspects of the medical domains (Treatment-Disease vs. mental health) and constructed using different methods:

- Caries Ontology (CO). CO was developed manually by a dentistry expert in our company.
- Disease-Treatment Ontology (DTO). We constructed an OWL translation of the ontology proposed by Khoo et al. [39].
- Mental Diseases Ontology (MDO). This ontology is publicly available.

#### Results of ontology validation

For the first step of the experiment, Table 4 presents the number of questions with respect to the number of classes, properties and instances of each ontology (DTO, MDO and CO) without question optimization.

**Table 4:** The number of ontology elements (OE) and the number of generated questions for different medical ontologies without optimization

Ontology	Number of classes	Number of properties	Number of instances	Total number of OE	Number of questions
DTO	49	148	0	197	165
MDO	149	76	18	243	243
CO	26	266	13	305	290

The number of generated questions depends on the ontology size and shows the importance of question ranking and optimization. The results indicate that the optimization method works better in case of ontologies with many instances. For the CO ontology, this strategy helps minimizing the number of submitted questions from 290 to 283 questions with only four NO answers. For the MDO ontology, our method allows asking 239 questions instead of 243 with only two NO answers. In case of ontologies with more NO answers (*i.e.* more invalid elements), the number of deleted questions will increase.

For the DTO ontology, the concepts have no instances and all facts were evaluated as correct by the expert, consequently the initial number of questions was conserved. The ontologies used in these experiments were constructed manually and semi-automatically. More experiments should be conducted on automatically constructed ontologies when available in order to evaluate more accurately the benefits of question optimization.

In the case of ontologies with few invalid elements (few NO answers), other methods should be used to optimize the presentation and reduce the time needed to answer the questions. For example, the following presentation methods can be studied: (i) question factorization according to an ontology element (concept, relation or individual) and (ii) logical chaining (A hasRelation1With B, B hasRelation2With C, etc.). Such organization can be effective in helping medical experts understand and answer the questions more quickly.

### Experiments on mapping validation

#### Materials and experimental procedure

We evaluate the NL quality of the questions generated automatically to validate mappings. We use two biomedical ontologies SNOMED-CT^9^ (SCT) and ICD-9-CM^10^ (ICD9) including different versions of official mappings established between them.

We aim to investigate to which degree it is possible to generate NL sentences that can adequately describe mappings. For this purpose, we evaluate the generated questions according to three standard measures in NL generation: grammaticality, fluency and meaning preservation. Since our approach aims to facilitate human intervention in mapping adaptation, we assume that it is relevant to assess the NL quality of the automatically-generated questions.

We presented the generated questions to three different human assessors who were asked to associate a score value between 1 and 10 for each dimension and each question. Assessors were ontology experts and familiar with the biomedical domain. We evaluated the approach for the validation of 20 randomly-selected adapted mappings generated from the evolution of mappings between SCT and ICD9.

We measure the Inter-Assessor Agreement (IAA) for grammaticality, fluency and meaning preservation. IAA corresponds to the average *κ* measure defined in [40]. The *κ* measure indicates how much the assessors’ agreement is above the probability of an agreement by chance, and it is commonly used in computational linguistics. In order to have relevant measures, we define 3 score intervals for grammaticality, fluency and meaning preservation which are: [0..3], [4..6], [7..10]. We use these intervals as categories in the calculation of the *κ* measure, which corresponds to:

2 $$ \kappa = \frac{P(a) - P(c)}{1 - P(c)}  $$

where *P*(*a*) refers to the observed inter-assessor agreement and *P*(*c*) is the probability of a chance agreement. *κ* values range from -1 to 1 (*cf.* Table 5 for results).

**Table 5:** Quality of the NL generated questions for mapping validation and average *κ* Inter-Assessor Agreement

	Grammaticality			Fluency			Meaning		
	Min.	Max.	Avg.	Min.	Max.	Avg.	Min.	Max.	Avg.
Assessor 1	0.4	1	0.775	0.4	1	0.81	0.4	1	0.915
Assessor 2	0.4	1	0.745	0.4	1	0.77	0.4	1	0.86
Assessor 3	0.6	0.9	0.735	0.6	0.9	0.745	0.6	0.9	0.77
Average *κ*		0.28			0.48			0.45	

#### Results of mapping validation

Table 5 presents the obtained results for the 20 Boolean questions that are generated for the 20 targeted mappings. They present the measures of IAA for grammaticality, fluency and meaning preservation.

Our second focus is to evaluate the usefulness of each question type. To this end, we count (i) the number of returned answer-types for the set of 20 questions according to the different question types and (ii) the number of validation/invalidation according to the observed adapted mappings (examining the evolution of the two official releases mappings in our dataset) (*cf.* Table 6). In this second independent evaluation, we asked the assessors to find a common agreement on the semantic correctness of the correspondences.

**Table 6:** Answer types according to question types

Answer type	Yes answers	No answers	Ambiguous
			source/target
Question type			
Boolean question	0 %	95 %	5 %
Revision of *c*_*s*_	0 %	95 %	5 %
Revision of *MR*	40 %	55 %	5 %
Revision of both	40 %	55 %	5 %
Final output validation	80 %	15 %	5 %

The overall assessment of the generated initial Boolean Questions (*cf.* Section “STEP 1: Boolean questions”) indicates good values for grammaticality and fluency because the attained average values are satisfactorily high regarding the used metric. The most important criterion for the mapping validation, which is meaning preservation, had the best score by the assessors. The *κInter-Assessor Agreement* is also relatively high (*κ* is not negative), which provides a positive test on the reliability of the assessors’ ratings. In short-term perspectives, further tests will be made with a correlation-based approach to have a more precise view on the levels of agreement.

Table 6 shows the percentage of different answer types returned during the validation of the adapted mappings. Results indicate that 80 % of the initial adapted mappings were validated or led to the validation of another new mapping (*i.e.*, re-adaptation) (*cf.* “Final output validation” row in table 6), discovered during the validation process. On the test set of 20 mapping adaptations, only one question was rated as ambiguous, due to an incomplete concept label. The low percentage of *Yes answers* for the initial Boolean questions indicates that the automatic selection of mapping adaptations using the approach described in [31] were insufficient for this dataset. The 3^*rd*^ type of question, revising only the mapping relation, allowed to validate 40 % of the mappings. Revising the source concepts alone fails to validate more mappings, but together with the revision of the mapping relation it allowed to validate 40 % of the mappings (*cf.* see 4^*th*^ type of questions “revision of both” in Table 6).

## Discussion and Future work

The experiments on ontology validation showed the need to add other specific types of questions and answer types. In some observed cases an answer can be *YES* but for a specific kind of patients (e.g. Infant) or also *NO* for a specific kind of patient, or under a specific condition. In our experiments the experts answered NO for such cases. Therefore, it would be interesting to give to the expert the possibility to specify a contextual element or an additional condition to their YES/NO answers. A possible solution can be to integrate factual questions as possible question type, which will also contribute to enrich the ontology during the validation process.

On the other hand, even if our optimization method may allow significantly reducing the number of questions, it is still challenging to validate very large ontologies with natural language questions. In this context, advanced content selection techniques such as summarization can play an important role. As suggested by Sure et al. [41], a summary of an ontology might include a couple of top levels in the ontology’s class hierarchy, and also the ontology’s hub concepts (*i.e.* concepts with the largest number of links).

To validate huge ontologies, we are considering approaches based on summarization. A possible solution can be by using the Key Concepts Extraction (KCE) algorithm which automatically extracts the most representative classes of an ontology. More particularly, summarization can be adapted to the validation task by taking into account several features such as the number of questions needed to validate a given extract or summary and the number of key concepts.

On the level of Mapping Validation, the conducted literature survey indicated that the generation of NL questions for mapping validation was not investigated. Our proposal originally evaluated the quality of generated NL questions to help human experts judge the quality of correspondences under evolution. The proposed method uses the context of the source concept to select similar concepts as alternatives in case of invalidated mappings.

In the conducted experiments on mapping validation, the analysis of quality-deficient Boolean questions produced by the NL generation system highlighted to two main error causes:

- The heterogeneity and length of the literal attributes that led to some inadequacies with the conceived patterns, e.g., “*Are other eye disorders more specific than family history degenerative disorder of macula?*”
- Errors in the concepts’ attributes (mainly labels), e.g. “ *Is other more specific than mechanical complication of suprapubic catheter?*”.

These observations show the importance of evaluating the linguistic quality of the ontology literals beforehand. This issue is particularly discussed in [42], where a meta-model is proposed to link ontology elements to relevant lexical entries. In the scope of our approach on question generation, an enhancement could be to (i) have several patterns that paraphrase the same mapping relation and (ii) syntactically parse the generated question to detect more trivial errors and choose alternative patterns if needed. According to our experiments, multiple choice questions with 5 correction alternatives for each invalid mapping proved to be efficient to locate the correct mapping relation and/or mapping target.

## Conclusions

The methods for automatic ontology construction have highlighted the problems of validation of ontologies and mappings. Research efforts and interests have grown more and more, but mostly at a formal level. In this article, we addressed the problem of ontology validation including mappings from a conceptual and a semantic point of view.

We proposed novel semi-automatic approaches based on the generation of questions and answers interpretation to facilitate the communication with domain experts. The defined methods generates natural language questions from medical ontologies and mappings and uses the answers from the expert to update/correct them.

Our approach implementing automatic methods might guide domain experts in the validation process. Relying on domain experts to monitor the validation can lead to various benefits, ensuring reliable communication between heterogeneous systems.

We evaluated the proposed methods based on several real datasets from different releases of biomedical ontologies and their associated mappings. The achieved results underscored the feasibility of our general approach for the validation of ontologies and mappings, and its relevant role in the completion of the their adequate evolution over time.

## Endnotes

^1^http://ihtsdo.org/snomed-ct/

^2^http://ncit.nci.nih.gov/

^3^http://bioportal.bioontology.org/

^4^http://www.chu-rouen.fr/cismef/

^5^ An international workshop is also held on question generation since 2008.

^6^http://nlp.stanford.edu/software/lex-parser.shtml

^7^http://www.w3.org/TR/rdf-mt/#RDFSRules

^8^ Free textual explanation is considered for future perspectives and is not used in this work.

^9^www.nlm.nih.gov/research/umls/licensedcontent/snomedctarchive.html

^10^www.cdc.gov/nchs/icd/icd9cm.htm
